# Supplementary material for: Plasma Extracellular Vesicles from Preeclamptic Patients Trigger a Detrimental Crosstalk Between Glomerular Endothelial Cells and Podocytes Involving Endothelin-1
Source: Int J Mol Sci. 2025 May 22;26(11):4962. doi: 10.3390/ijms26114962 (PMC12154044; doi:10.3390/ijms26114962)
Supplement: Supplementary file 1 [file ijms-26-04962-s001.zip › ijms-3489804-supplementary.pdf]

SUPPLEMENTARY FILE

Methods

Cell cultures

Glomerular endothelial cells (GEC) and podocytes (PODO) were obtained from normal cortex fragments of surgically removed human kidneys (nephrectomy due to cancer). After isolation by cell sorting, GEC and PODO were immortalized by infection with a hybrid Adeno5/SV40virus. GEC were cultured on gelatin-coated flasks using EBM medium containing endothelial growth factors (Lonza, Basel, Switzerland), whereas PODO in Dulbecco's Modified Eagle Medium (DMEM from Euroclone S.p.A., Milan, Italy). All media contained 2 mM L-glutamine (Euroclone), 1% penicillin and streptomycin and 10% fetal bovine serum (Euroclone). For experimental procedures, GEC and PODO were plated in 96-well plates (Falcon Labware, Oxnard, CA, USA) at a concentration of 10000 cells/well and stimulated with EVs derived from plasma of PE patients or HCs, performing the assays described below.

Cell viability assay

Briefly, 10000 cells/well (GEC or PODO) were stimulated with different types of plasma-derived EVs for 24 h (50000 EVs/cell), whereas non-stimulated cells were used as control. At the end of stimulation, cell viability was examined by using the 1% 3-[4,5-dimethylthiazol-2-yl]-2,5-diphenyl tetrazolium bromide dye (MTT from Life Technologies, Monza, Italy). After treatment, medium was removed and 0.5 mg/mL MTT dye was added to each well and incubated for 2 h at 37°C. Cell viability was determined by measuring the absorbance through a spectrometer (VICTOR™ X Multilabel Plate Reader; PerkinElmer) at a wavelength of 570 nm. Cell viability was calculated by setting control cells as 100%. All experiments were conducted in triplicate.

ROS production

After stimulation with different plasma-derived EVs, 5-(and-6)-carboxy-2′,7′-dichlorodihydrofluorescein diacetate (carboxy-H2DCFDA) was added to GEC or PODO according to manufacturer's instructions (Image-iT LIVE Green ROS Detection Kit, Life Technologies). After 30 min cells were analyzed by FACS or immunofluorescence. ROS release was analyzed by reading the excitation and emission wavelength at 480 nm and 560 nm, respectively, through a spectrophotometer (VICTOR™ X Multilabel Plate Reader). Normalization of the data was executed vs. untreated cells (control). All experiments were conducted in triplicate.

NO Release by GEC

In GEC, NO release was evaluated by Griess method (Promega Italia S.r.l., Milan, Italy): for this experimental procedure, 10,000 cells/well were cultured in 96-well plates in complete culture medium and then incubated with EVs isolated from plasma of PE patients or HCs. After stimulation, NO production in supernatants was examined by adding an equal volume of the Griess reagent following manufacturer's instruction. The reading of each sample was performed at 570 nm through a spectrometer (VICTOR™ X Multilabel Plate Reader). A standard curve was prepared to quantify NO production, which was expressed as nitrites (μM). All experiments were conducted in triplicate.

Albumin diffusion assay

The percentage of albumin diffusion was also evaluated by a colorimetric assay. Briefly, GEC or PODO (10000 cells/well) were grown in a complete medium on 0,4 μm pore size Transwell inserts in a 24-well culture plate (Euroclone) to form a confluent monolayer. After reaching confluence, medium was removed and the monolayer was immediately covered with 500 μL DMEM (free of FBS and phenol red) containing 5% FITC-conjugated albumin and EV isolated from plasma of PE patients or HCs (50000 EV/cell). After EV stimulation, albumin-FITC diffusion across the monolayer was quantified by measuring absorbance at 590 nm (VICTOR™ X Multilabel Plate Reader; PerkinElmer). In 3 different experimental procedures, following EV stimulation, albumin diffusion was compared with matched controls (non-stimulated cells) and expressed as:

$$\text{\% of diffusion} = \frac{\text{mean Abs}_{590\ nm}\ \text{sample} - \text{mean Abs}_{590\ nm}\ \text{control}}{\text{mean Abs}_{590\ nm}\ \text{control}} \times 100$$

Endothelial-to-Mesenchymal transition (EndMT) in GEC

To evaluate EndMT, 10000 GECs/well were stimulated with different EV types as previously described for the other assays. After treatment, GEC were washed with saline and detached with low-concentrated trypsin (0.01X)-EDTA in 1x PBS ice cold. We assessed surface markers by FACS analysis using allophycocyanin-conjugated (APC) anti-CD31 (Miltenyi Biotec, Bergisch Gladbach, Germany), phycoerythrin-conjugated (PE) anti–vascular endothelial (VE) Cadherin (Miltenyi Biotec) and cytoplasmic markers. Intracellular staining was characterized by fixation and permeabilization with IntraPrep Kit (Instrumentation Laboratory, Bedford, MA, USA) before continuing with conjugated antibody staining, such as PE-conjugated anti-vimentin (Miltenyi Biotec) and FITC-conjugated anti–type I collagen (Millimark; MilliporeSigma). After washing, cells were incubated with FcR blocking reagent (Miltenyi Biotec) for 10 min at room temperature. After blocking, primary antibodies were added for 25 min at 4°C, and then cells were washed with the FACS buffer and re-suspended in each tube with 500 µl of FACS buffer for FACS analysis. Data were obtained using FC500 flow cytometer (Beckman Coulter, Brea, CA, USA). Quantification was determined by using an isotype-matched mAb. A total of 3 independent experiments were performed.

Nephrin expression by PODO

To evaluate nephrin expression in PODO, 50000 cells/well were plated and stimulated with PE- or HC-derived plasma EVs. After incubation, detached cells were resuspended with 100 μL of sterile saline to make a final concentration of $1*10^6$ cells/mL. Thereafter, 10 μg/mL of FITC-conjugated antibody directed to human nephrin (Santa Cruz Biotechnology) was added for 1 h at 4°C in dark conditions. Analysis was performed by using Attune™ NxT flow cytometer (Thermo Fisher Scientific). All experiments were conducted in triplicate.

GEC and PODO co-culture model

In selected experiments, a co-culture GEC-PODO model was developed with the aim to study in more details the crosstalk occurring in the glomerular filtration barrier. In this model, GEC or PODO was initially plated in 96 well plates and incubated with EVs derived from plasma of PE patients or HCs as described for the previous assays. After EV stimulation, supernatants from GEC were used to stimulate PODO, whereas supernatants from PODO were used to stimulate GEC. For both conditions, experiments were conducted in 96 well plates and cells were stimulated with 100% medium collected from the other cell line. Following supernatant incubation, the following assays were performed on GEC or PODO with the same procedures described above: MTT, ROS release and albumin diffusion. Moreover, we investigated the role of the Endothelin-1 receptor antagonist PD142893 (10 μM) on nephrin expression (FACS analysis) and on permeability to albumin on PODO stimulated with supernatants of GECs previously incubated with plasma EVs.
